# Accreditation and qualification of primary care teaching practices in Germany – a nationwide online survey of universities

**DOI:** 10.3205/zma001848

**Published:** 2026-04-15

**Authors:** Sabine Gehrke-Beck, Isabel Kitte, Irmgard Streitlein-Böhme, Tobias Deutsch, Iris Demmer, Maryna Gornostayeva, Ralf Jendyk

**Affiliations:** 1 Charité – Universitätsmedizin Berlin, corporate member of Freie Universität Berlin and Humboldt-Universität zu Berlin, Institut für Allgemeinmedizin, Berlin, Germany; 2 Charité – Universitätsmedizin Berlin, Ambulantes Gesundheitszentrum AGZ, Berlin, Germany; 3 Medizinische Hochschule Hannover, Institut für Allgemeinmedizin und Palliativmedizin, Hannover, Germany; 4 Gesellschaft für Hochschullehre in der Allgemeinmedizin, Berlin, Germany; 5 Ruhr-Universität Bochum, Medizinische Fakultät, Abteilung für Allgemeinmedizin, Bochum, Germany; 6 Universität Leipzig, Medizinische Fakultät, Institut für Allgemeinmedizin, Leipzig, Germany; 7 Universitätsmedizin Göttingen, Medizinisches Versorgungszentrum, Göttingen, Germany; 8 Medizinische Fakultät Mannheim der Universität Heidelberg, Zentrum für Präventivmedizin und Digitale Gesundheit (CPD), Abteilung Allgemeinmedizin, Mannheim, Germany; 9 Deutsche Gesellschaft für Allgemeinmedizin (DEGAM), Sektion Studium und Hochschule, Berlin, Germany; 10 Universitätsmedizin Rostock, Institut für Allgemeinmedizin, Rostock, Germany

**Keywords:** preceptorship, clinical clerkship, teaching qualification, primary care, undergraduate medical education

## Abstract

**Objectives:**

Compile information on the current measures undertaken by German universities to accredit and qualify primary care teaching practices.

**Methods:**

Nationwide online survey of teaching practice coordinators using a self-developed questionnaire, descriptive analysis of the quantitative data and qualitative content analysis of the free-text responses.

**Results:**

A total of 32 out of 41 university sites provided information. A structured accreditation process is conducted at 29 sites, most commonly by personally visiting the medical practices (n=22), alternatively through video calls (n=10) or telephone calls (n=9). 18 sites have a process in place to qualify medical practices for the block practicum (obligatory for qualification at 15 of these sites), 17 sites have one for the practical year (obligatory at 12), and 22 sites offer other additional training (mandatory at 8). Procedural formats and length of time vary. The imparted content includes organizational information, targeted learning objectives, and teaching methods.

**Conclusion:**

Structured accreditation and qualification measures are carried out by many universities. A nationwide harmonization of teaching practice qualification is advantageous for enabling cross-site qualification programs.

## Zusammenfassung

**Ziele:**

Erfassung der aktuellen Akkreditierungs- und Qualifizierungsmaßnahmen für allgemeinmedizinische Lehrpraxen an den universitären Standorten in Deutschland.

**Methoden:**

Bundesweite online Befragung der Lehrpraxenkoordinator:innen mit selbst entwickeltem Fragebogen, deskriptive Auswertung der quantitativen Angaben, qualitative Inhaltsanalyse von Freitextangaben.

**Ergebnisse:**

32 von 41 Standorte machten Angaben. An 29 Standorten wird eine strukturierte Akkreditierung durchgeführt, am häufigsten durch persönliche Praxisbesuche (n=22), alternativ durch Videocalls (n=10) oder Telefonate (n=9). An 18 Standorten existieren Qualifizierungsangebote für das Blockpraktikum (an 15 davon verpflichten), an 17 Standorten für das PJ (an 12 davon verpflichtend) und an 22 Standorten weitere ergänzende Angebote (an 8 davon verpflichtend). Formate und Dauer variieren. Inhaltlich werden organisatorische Informationen, angestrebte Lernziele und didaktische Methoden vermittelt.

**Schlussfolgerung:**

An vielen Standorten werden strukturiert Akkreditierungs- und Qualifizierungsmaßnahmen durchgeführt. Eine bundesweite Abstimmung der Lehrpraxenqualifizierung ist vorteilhaft, um standortübergreifende Qualifizierungsangebote zu ermöglichen.

Republication of: Gehrke-Beck S, Kitte I, Streitlein-Böhme I, Deutsch T, Demmer I, Gornostayeva M, Jendyk R. Accreditation and qualification of primary care teaching practices in Germany – a nationwide online survey of universities. GMS J Med Educ. 2025;42(5):Doc56. DOI: 10.3205/zma001780

## Research question

How are primary care practices accredited and qualified for teaching in Germany?

## Introduction

In Germany, medical students receive practical training in general practice through one-on-one teaching at primary care practices. To make this possible, the universities work with so-called networks of academic teaching practices specialized in general practice. It is known that general practice internships can positively influence students’ interest in pursuing a career in primary care, whereby quality of teaching is a significant factor, too [1], [2]. Recommendations have been given in agreed position papers for both the accreditation and the didactic qualification of teaching practices [3], [4].

This study examines the status of implementation at universities across Germany in 2024. The results serve as a starting point for a newly formed working group of the German Society of University Teachers of General Practice (GHA) and the German Society for General Practice and Family Medicine (DEGAM) with the aim of further developing accreditation and qualification measures.

## Methods

A cross-sectional study using a self-developed, online questionnaire was conducted via SoSciSurvey [https://www.soscisurvey.de/]. Data was collected through closed questions and supplemental free-text responses regarding the number of teaching practices and their functions, the need and methods for recruiting new teaching practices, accreditation criteria and procedures, and qualification programs. The survey was sent to the working group members on October 23, 2023, (reminder on January 9, 2024) and to the GHA representatives at the other nationwide sites on January 7, 2024 (reminder on January 17, 2024); the survey could be completed up until February 15, 2024. Descriptive statistical analysis followed using SPSS along with structuring qualitative content analysis [5] of the free-text responses.

## Results

A total of 32 out of 41 university sites participated in the survey. Between 60 and 538 teaching practices are involved in educating medical students per site (9 sites <150 teaching practices, 14 sites 150-250 teaching practices, and 7>250 teaching practices, 2 no data). These teaching practices train and instruct students in the general practice block practicum (BP) (n=32, 1 no data) and in the general practice clinical elective during the practical year of undergraduate medical study (PJ) (n=30, 2 sites are newly founded medical schools and have not yet offered a fifth year of practical study). At 17 sites the teaching practices provide instruction to medical students in the elective subjects, at 15 sites as part of job shadowing. At individual sites, the teaching physicians also give instruction in local academic projects or give lectures and seminars.

### Recruiting

According to the respondents, there are usually enough accredited teaching practices at their sites to ensure the undergraduate education required by the current medical licensing regulations governing the BP and the practical year (a sufficient number of teaching practices is present at each of n=31 sites). New teaching practices are recruited through informative meetings (n=22 sites), the institute or department homepage (n=18), and written letters (n=8). Also mentioned in the free-text responses were local networks (n=4), making personal contact (n=3), and recruiting events (n=2). Two sites actively ask students for their recommendations about suitable practices.

### Accreditation

A standardized accreditation process has been defined at 29 sites. At 16 sites, teaching practices are accredited according to criteria set by GHA, DEGAM and the Association for Medical Education (GMA) [4]. Accreditation takes place in the form of personal visits to the medical practices (n=22), telephone calls (n=9) and/or video calls (n=10).

### Qualification programs

There are programs for BP qualification at 18 sites, with required attendance at 15 of these sites. A similar number of programs exist to qualify for the practical year (n=17) and are somewhat less often mandatory (12 von 17). Supplemental offerings, such as regularly scheduled meetings for the teaching physicians, take place at 22 sites, of which attendance is required at only 8 of them. The process of qualification extends over different time periods, online or in-person or in a combination of the two (see table 1 (Tab. 1)).

Table 2 (Tab. 2) summarizes the content contained in the free-text responses. It is seen here that organizational aspects (including the legal framework affecting the practical year), learning objectives, and didactic methods in the practice setting play a role. Training to become an examiner for the state medical exam (M3) is also offered as part of the qualification for the practical year. Additional offerings impart didactic theory and methodology aimed at teaching inside and outside of the practice setting.

## Discussion

The survey shows that most of the universities follow a structured approach to accrediting and qualifying medical teaching practices. The frequency with which qualifications are carried out had been documented already in 2016, prior to drafting the position paper [3]. At that time, 30 of 37 sites participated: 18 conducted BP qualification (obligatory at 14), 15 conducted qualification for the practical year (obligatory at 9). An increase is visible primarily in qualifications for the practical year, possibly in preparation for a required quarter of general practice that would be included under new medical licensing regulations. The duration of the current courses is for the most part much shorter than envisioned in the 2020 position paper [3]. Furthermore, 10 sites do not carry out any qualification measures. Personal follow-ups regarding this cited mainly constraints on human resources as a barrier to implementation.

Despite the heterogeneity in regard to how qualification is carried out, overlaps between the sites were found in the survey responses, such that the working group aims to reach a nationwide consensus on a core curriculum for BP and practical year qualifications. This enables cross-site programs and mutually recognized qualifications, thereby supporting sites with fewer human resources.

To our knowledge, no data has been systematically collected concerning the qualification of teachers to instruct students in the internships in other specialties. Rather, for these there are only isolated offerings and hardly any structured or mandatory programs. Specific teaching qualifications for clinical internships could also boost the quality of teaching in other specialties and, potentially, be easy to implement in the hospital setting if courses would take place in the workplace during working hours.

Among the survey’s limitations is the possibility that we received responses specifically from sites with structured processes for accrediting and qualifying medical teaching practices. Also, it was not documented in detail how the accreditation criteria were defined and verified. Our survey only covered the measures to accredit and qualify teaching practices. However, comprehensive quality assurance also entails regular evaluations, a structured complaints management system, and a defined procedure for handling incidents such as discrimination or harassment. These aspects should also be examined and agreed upon nationally.

## Conclusions

Primary care teaching practices are for the most part accredited in a structured manner, and at many sites physician teachers are often required to obtain a qualification in order to teach undergraduate medical students. A nationwide consensus on content and formats can enable cross-site training and mutually recognized qualifications, as well as relieve the strain on resources at individual sites.

## Notes

### Ethical approval and data protection

This study did not involve any patients or students. In the survey it was possible to voluntarily give the names of contact persons at a particular site; no personal data was otherwise collected. The names of the contact persons were only used for communication within the working group and with the people responsible for the teaching in general practice. The survey respondents were informed about the handling of this data and participated voluntarily. No personal data are used in the publication; all of the data and information have been summarized and are not presented in a site-specific manner. All of the participating sites were informed about the intention to publish. Ethical approval was not obtained because no patients or students were involved and no personal data were used for publication.

### Authors’ ORCIDs

- Sabine Gehrke-Beck: [0000-0002-6221-2813]
- Iris Demmer: [0000-0001-9652-9803]
- Ralf Jendyk: [0000-0002-2776-0515]

## Acknowledgements

We wish to thank all of the GHA representatives and the teaching coordinators who participated in the survey.

## Competing interests

The authors declare that they have no competing interests. 

## Versioning

This article is the corrected version of an article originally published in issue 5 / 2025 of the journal [6], [7].

## Figures and Tables

**Table 1:**
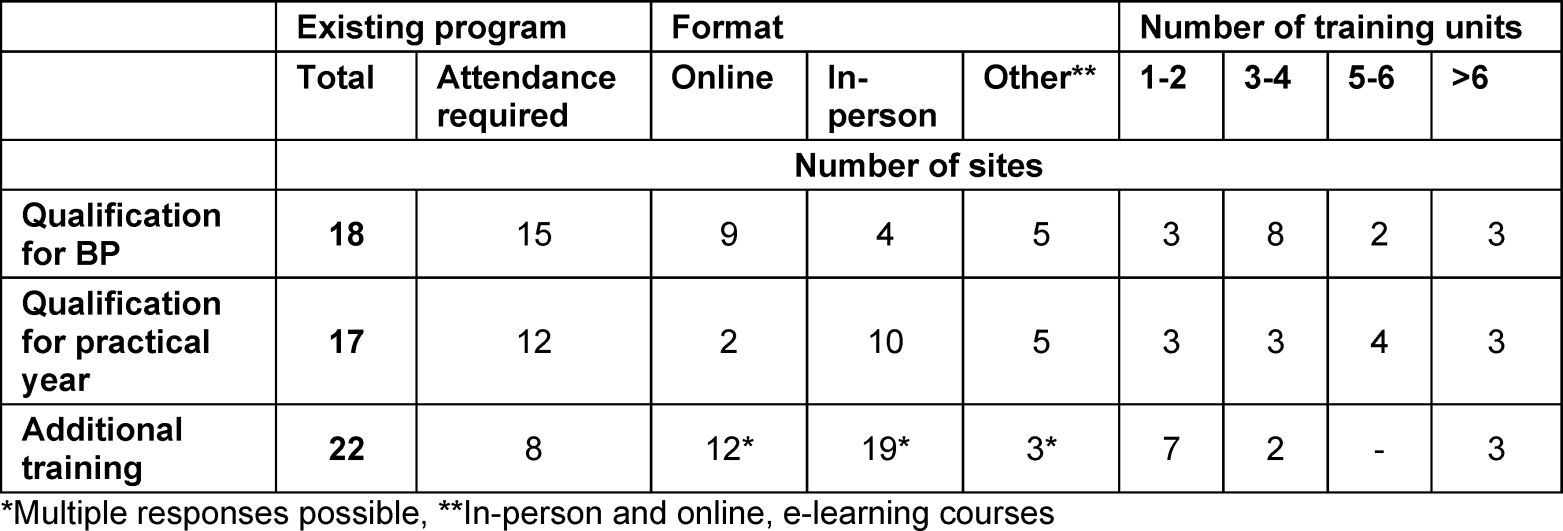
Training programs/courses offered by the universities to qualify primary care physicians to teach medical students

**Table 2:**
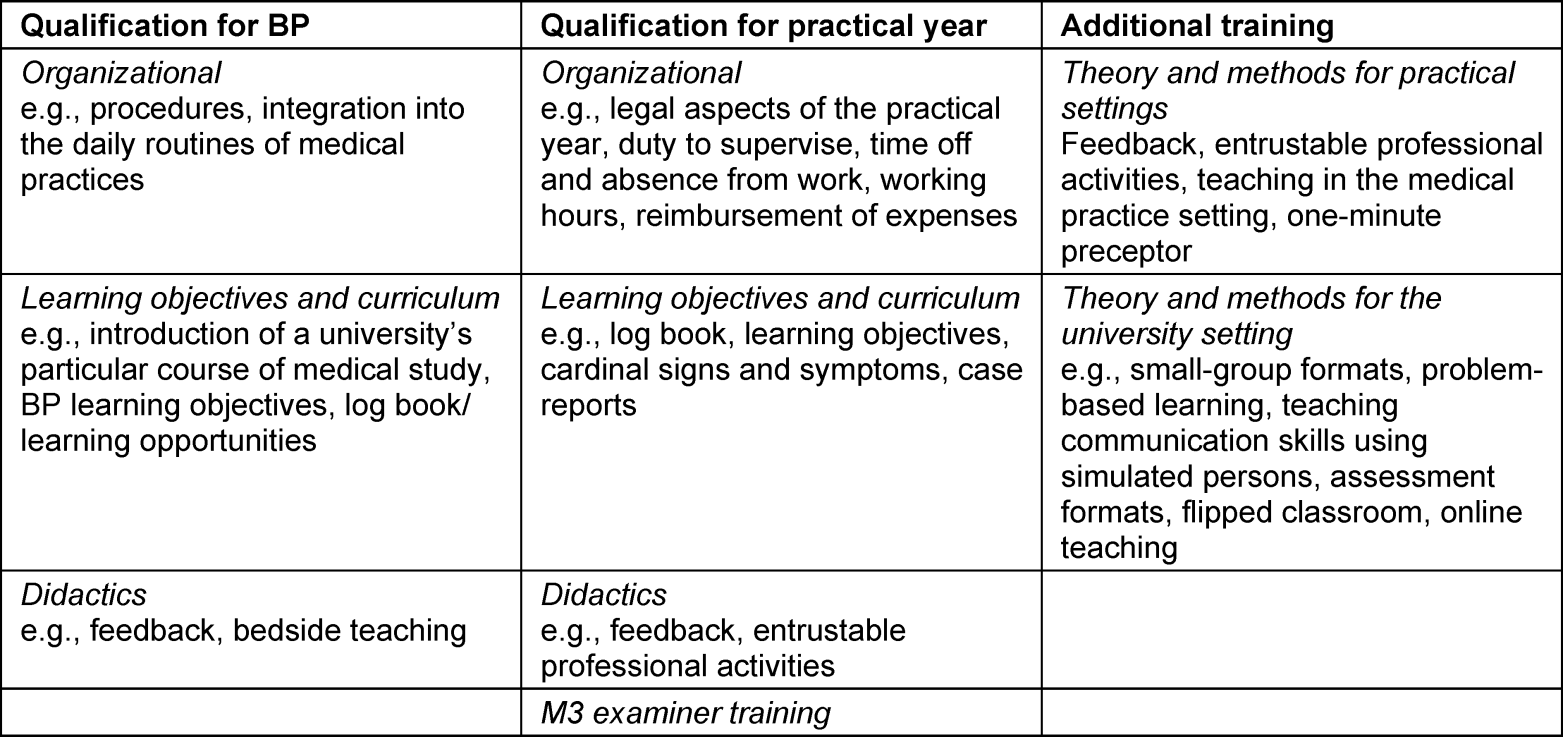
Content of the free-text responses on the qualification programs/courses for primary care teaching physicians
